# Investigating the shared genetic architecture between selective immunoglobulin A deficiency and autoimmune diseases

**DOI:** 10.1007/s00439-026-02850-5

**Published:** 2026-06-22

**Authors:** Xiao Dang, Frank Qingyun Wang, Caicai Zhang, Huidong Su, Yao Lei, Jing Yang, Yu Lung Lau, Wanling Yang

**Affiliations:** Department of Paediatrics and Adolescent Medicine, The University of Hong Kong, Hong Kong, China

## Abstract

Selective immunoglobulin A deficiency (IgAD) is the most prevalent primary immunodeficiency and frequently coexists with autoimmune diseases (ADs), suggesting a shared genetic etiology. While genome-wide association studies (GWAS) have identified only a few risk loci for IgAD and hundreds for ADs, systematic cross-trait analyses are lacking, leaving the shared genetic architecture and underlying mechanisms poorly understood. In this study, we analyzed summary statistics from large-scale GWAS of IgAD and 10 common ADs. Cross-trait GWAS meta-analysis identified 51 pleiotropic loci significantly associated with IgAD and at least one AD (p < 5×10⁻⁸), 31 of which were novel for IgAD. Colocalization analysis further supported 33 shared loci between IgAD and ADs, including 19 novel IgAD loci, such as those near *TNFAIP3*, *CD28*, *IRF4*, *STAT4*, *SH2B3*, *APOBR* and *RAD51B*. Gene-level analysis revealed shared pathways involved in T-cell differentiation and the intestinal immune network for IgA production. Tissue heritability analysis identified whole blood and the intestine as critical tissues, while single-cell RNA sequencing (scRNA-seq) highlighted B cell subtypes in the peripheral blood and gut as the most affected cell types in IgAD and ADs. Mendelian randomization further demonstrated bidirectional causal relationships between IgAD and several ADs. Our findings reveal a shared genetic architecture between IgAD and ADs, highlighting common functional mechanisms and providing insights into their biological interplay and potential immune-based therapies.

**Supplementary Information:**

The online version contains supplementary material available at 10.1007/s00439-026-02850-5.

## Introduction

Selective immunoglobulin A deficiency (IgAD) is the most common primary immunodeficiency, characterized by markedly reduced serum levels of immunoglobulin A (IgA) while maintaining normal levels of other immunoglobulin isotypes (Yel 2010). The prevalence of IgAD varies across populations, affecting approximately 1 in 3,000 to 1 in 150 individuals, with the highest incidence observed in those of European descent (Vosughimotlagh et al. 2023). Genetic factors are believed to play a significant role in the pathogenesis of IgAD, as familial inheritance is observed in approximately 20% of cases (Karaca et al. 2018), suggesting a heritable component. Evidence also points to autosomal transmission with variable penetrance, further highlighting the complexity of its genetic basis (Schäffer et al. 2006). Genome-wide association studies (GWAS) have identified several significant loci associated with IgAD, offering valuable insights into the underlying genetic architecture and the etiology of this disease (Bronson et al. 2016).

Most individuals with IgAD are asymptomatic (Yel 2010; Vosughimotlagh et al. 2023). When symptoms are present, the most common clinical manifestations include infections, followed by allergic diseases and autoimmune conditions (Vosughimotlagh et al. 2023). Notably, autoimmune diseases (ADs) are present in approximately 36% of patients with IgAD (Amaya-Uribe et al. 2019). The most prevalent ADs—listed in descending order of frequency—are celiac disease (CD), inflammatory bowel disease (IBD), rheumatoid arthritis (RA), systemic lupus erythematosus (SLE), and type 1 diabetes (T1D) (Vosughimotlagh et al. 2023). These conditions occur at significantly higher rates in individuals with IgAD compared to the general population.

Conversely, studies have also reported decreased levels of IgA in patients with ADs. For instance, an increased frequency of IgAD has been observed among patients with SLE, with reported rates ranging from 1:130 in Spain to 1:19 in the USA (Wang et al. 2011). Similarly, elevated prevalence rates of IgAD have been noted in patients with T1D, ranging from 1:261 in the USA to 1:27 in Italy (Wang et al. 2011). Furthermore, IgAD shows a strong association with CD, with an overall prevalence of 1:39, and RA, with a prevalence of 1 in 194 across populations (Wang et al. 2011). This bidirectional epidemiological overlap suggests that the co-occurrence of IgAD and ADs is unlikely to be incidental and may instead reflect shared biological and genetic susceptibility. Moreover, patients with IgAD frequently exhibit broader immune dysregulation, including the presence of autoantibodies and increased autoimmune manifestations, further supporting a link between immunodeficiency and autoimmunity (Odineal and Gershwin 2020).

Although the relationship between IgAD and autoimmunity is well-documented, the underlying causes of this association remain poorly understood. Mechanistically, it has been suggested that a failure in switched memory B cells may contribute to both IgAD and autoimmunity (Aghamohammadi et al. 2011; Grosserichter-Wagener et al. 2020). At the genetic level, a shared predisposition has been proposed to explain the co-occurrence of these conditions (Bronson et al. 2016; Singh et al. 2014). GWAS have identified several significant loci associated with IgAD, including HLA locus genes (*HLA-DRB1*, *HLA-DQB1* and *HLA-DQA1*), as well as *IFIH1*, *PVT1*, *AHI1* and *CLEC16A* (Bronson et al. 2016). However, previous studies have largely focused on IgAD alone or on individual autoimmune conditions, and a comprehensive investigation of the shared genetic architecture, pleiotropic loci, common biological pathways, relevant tissues/cell types, and potential causal relationships between IgAD and a broad spectrum of ADs remains limited.

A systematic cross-disease genetic analysis may therefore provide important insight into the pathogenesis of IgAD. By identifying genetic variants and biological pathways shared between IgAD and ADs, such an approach can help explain why these conditions co-occur, distinguish common from disease-specific mechanisms, and prioritize candidate genes, tissues, and cell types involved in their overlap. It may also clarify whether the relationship between IgAD and ADs reflects shared polygenic susceptibility, local pleiotropy, or potential causal effects.

**Fig. 1:**
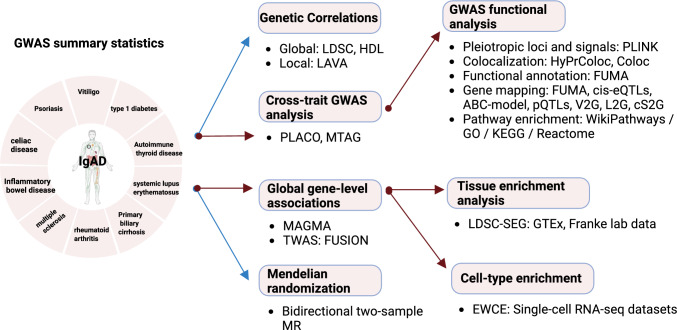
Global overview of the study. The workflow summarizing the study design and methodology was created using BioRender (https://biorender.com/).

In this study, we performed comprehensive analyses and applied advanced statistical methods to investigate the genetic overlap between IgAD and autoimmunity. We used global and local genetic correlations, cross-trait GWAS meta-analysis, and multi‑trait colocalization analysis to identify novel pleiotropic loci underlying IgAD and autoimmune conditions. Additionally, we conducted functional annotation and gene mapping of the shared loci. To further explore shared biological functions and cellular components implicated in IgAD and ADs, we leveraged single-cell RNA sequencing data and applied cell-type enrichment analysis. This study enhances our understanding of the underlying mechanisms linking IgAD and autoimmunity. The identification of pleiotropic loci provides deeper insight into the genetic underpinnings of both conditions and opens new avenues for the development of targeted therapies. A systematic flowchart of the study design is shown in Fig. 1.

## Methods

### GWAS summary statistics source

GWAS summary statistics for IgAD and the 10 most common ADs were obtained from publicly available datasets in the NHGRI-EBI GWAS catalog (https://www.ebi.ac.uk/gwas/). These included traits were: IgAD (Bronson et al. 2016), Celiac disease (CD) (Trynka et al. 2011), Inflammatory bowel disease (IBD, including Crohn's disease and ulcerative colitis) (De Lange et al. 2017), Multiple sclerosis (MS) (International IBD Genetics Consortium (IIBDGC), et al. 2013), Primary biliary cirrhosis (PBC) (Cordell et al. 2021), Common Psoriasis (PV, including psoriasis vulgaris) (Tsoi et al. 2012), Rheumatoid arthritis (RA) (Ishigaki et al. 2022), Systemic lupus erythematosus (SLE) (Wang et al. 2021), Type 1 diabetes (T1D) (Chiou et al. 2021), Vitiligo (VIT) (Jin et al. 2016) and Autoimmune thyroid disease (ATD) (Saevarsdottir et al. 2020). We focused mainly on studies involving populations of European ancestry for which summary statistics were available, selecting the study with the largest cohort size for each trait. Detailed sources and accession numbers for the included studies are summarized in **Supplementary Table ****1**. To ensure consistency, we applied the same quality control procedures across all studies. Summary statistics were restricted to variants from the 1000 Genomes Project Phase 3 European reference panel, and rare variants with a minor allele frequency (MAF) below 0.01 were excluded (Auton et al. 2015).

### Identifying global genetic correlations

We employed both linkage disequilibrium score regression (LDSC) (Bulik-Sullivan et al. 2015) and high-definition likelihood (HDL) (Ning et al. 2020) to assess the global genetic correlation (r_g_) between IgAD and the 10 ADs using GWAS summary statistics. LDSC leverages the expected relationship between linkage disequilibrium (LD) and GWAS association statistics to estimate heritability and assess genetic overlap between traits (Bulik-Sullivan et al. 2015). For LDSC analysis, we used pre-computed LD scores from the European reference panel in 1000 Genomes Project Phase 3 and restricted the analysis to variants within well-imputed HapMap3. HDL is a likelihood-based method for estimating genetic correlation between traits. For HDL analysis, we utilized a reference panel of 1,029,876 quality-controlled UK Biobank imputed HapMap3 SNPs, pre-computed for populations of European ancestry (Ning et al. 2020). Finally, to control for multiple testing, we applied the false discovery rate (FDR) correction to the results obtained from both methods.

### Evaluating local genetic correlations

While global genetic correlations provide an average measure of shared association across the genome, inconsistencies in the directions of genetic correlations in different regions may result in nonsignificant global estimates. To address this, we applied LAVA (Werme et al. 2022) to assess pairwise local genetic correlations between IgAD and the 10 ADs across independent genomic regions. LD was estimated using the 1000 Genomes Project Phase 3 European reference panel. Local genetic correlations were evaluated across 2,495 independent genomic regions and the FDR correction was applied to account for multiple testing.

### Cross-trait GWAS meta-analysis

We conducted cross-trait GWAS meta-analyses using two independent approaches based on GWAS summary statistics to identify shared risk variants between IgAD and each of the ADs. (1) PLACO (pleiotropic analysis under composite null): PLACO identifies pleiotropic variants between two traits in population-based studies by testing the null hypothesis that at most one trait is associated with a given SNP, against the alternative hypothesis that both traits are associated (Ray et al. 2021). (2) MTAG (multi-trait analysis of GWAS): MTAG performs a meta-analysis of GWAS summary statistics across different traits, allowing for shared genetic signals while being robust to sample overlap (Turley et al. 2018). Cochran’s Q test was performed via METAL (Willer et al. 2010) to estimate the heterogeneity of effect sizes for shared genetic variants between IgAD and each AD.

### Identification of pleiotropic loci and signals

Independent SNPs were identified using the PLINK (Purcell et al. 2007)(v1.9) clumping function with the following parameters: --clump-p1 5e-8 --clump-p2 1e-5 --clump-r2 0.2 --clump-kb 250. We used the 1000 Genome Project Phase 3 European reference panel to calculate pairwise LD between SNPs. To avoid potential bias caused by the extreme and complex LD structure in the major histocompatibility complex (MHC) region, SNPs within the MHC region (chr6: 25,000,000-35,000,000) were excluded from the downstream analyses. Within each locus, the variant with the lowest P value was defined as the lead SNP, and candidate tag SNPs were defined as all SNPs with an r2 > 0.2 relative to the lead SNP. Independent loci were designated by extending a ±250kb region around each lead SNP, with overlapping loci merged into a single genomic locus. Subsequently, independent loci across all IgAD-AD pairs were merged into distinct loci by unifying their genomic boundaries if loci from different IgAD-AD pairs overlapped. While the lead SNPs from each IgAD-AD pair were retained, they were grouped into association signals based on LD (r2 > 0.2) within the merged locus.

To ensure the robustness of our findings and identify additional shared-risk SNPs, we defined significant pleiotropic variants as lead SNPs meeting the following criteria in the cross-trait meta-analysis: P < 5×10^-8^ using PLACO and P < 1×10^-5^ using MTAG for IgAD and each of the ADs. A locus was considered novel if all candidate SNPs within the locus had not been previously reported as significantly associated with IgAD in the GWAS Catalog or other literature, and the SNP did not reach the genome-wide significance threshold (P < 1×10^–5^) in the original IgAD GWAS summary statistics. For visualization, regional locus zoom plots were generated using LocusZoom (Pruim et al. 2010).

### Multi‑trait colocalization analysis

We used the HyPrColoc (Foley et al. 2021) and Coloc (Giambartolomei et al. 2014) R packages to perform colocalization analysis, aiming to determine whether the pleiotropic loci identified by PLACO and MTAG were shared between IgAD and ADs within the same genomic locus. HyPrColoc is a Bayesian divisive clustering algorithm that utilizes GWAS summary statistics to detect colocalization across multiple traits simultaneously (Foley et al. 2021). Coloc package performs pairwise colocalization analysis to evaluate whether two traits share common genetic causal variant(s) within a given locus (Giambartolomei et al. 2014). We conducted the colocalization analysis between IgAD and each AD in each identified pleiotropic locus. A locus was considered colocalized if the posterior probability (PP) from HyPrColoc exceeded 0.5, or the PP.H4 (indicating both traits are associated and share a single causal variant) from Coloc exceeded 0.5.

### Functional annotation and gene mapping analysis

We employed the FUMA (Watanabe et al. 2017) online platform (https://fuma.ctglab.nl/) to perform functional annotation for lead SNPs identified through PLACO and MTAG. FUMA provides comprehensive functional annotations, including ANNOVAR analysis, CADD scores, and RegulomeDB scores. CADD scores predict the likelihood of a variant being pathogenic while RegulomeDB scores assess the regulatory functionality of SNPs.

Following functional annotation, SNPs were mapped to putatively functional genes using multiple algorithms: (1) Mapping SNPs to genes located within a 10kb window or to genes predicted to be affected based on ANNOVAR, a process implemented directly within FUMA. (2) Mapping SNPs to significant cis-eQTL (FDR<0.05) genes derived from six large-scale studies focused on immune cells, namely DICE (Schmiedel et al. 2018) (13 immune cells and two activated cell types), ImmuNexUT (Ota et al. 2021) (28 immune cell types), GTEx (G 2020) (whole blood, spleen, and EBV-transformed lymphocytes), BLUEPRINT (Kundu et al. 2022) (monocytes, neutrophils, CD4+ T cells), eQTLGen (Võsa et al. 2021) (whole blood), and scRNA-seq of ADs from the OneK1K study (Yazar et al. 2022) (14 immune cell types); (3) Mapping SNPs to genes based on Activity-By-Contact(ABC) model (Nasser et al. 2021), an enhancer-gene linking approach utilizing data from blood cells to predict regulatory interactions; (4) Mapping SNPs to plasma protein levels (pQTLs) using data from the UK Biobank (Sun et al. 2023) and Icelandic studies (Eldjarn et al. 2023); (5) Mapping SNPs to genes using integrated SNP-to-gene linking strategies from three tools: V2G (Ghoussaini et al. 2021) and L2G (Mountjoy et al. 2021) from the Open Targets Platform, as well as cS2G (Gazal et al. 2022), which combines multiple evidence sources to prioritize causal genes.

### Functional pathway enrichment analysis

Functional enrichment analysis was conducted using the R package clusterProfiler to identify biological pathways and processes associated with the candidate genes. The analysis used terms from WikiPathways, Gene Ontology (GO), Kyoto Encyclopedia of Genes and Genomes (KEGG), and Reactome databases (Yu et al. 2012). Enrichment terms with an adjusted p-value < 0.05 were considered statistically significant.

### Tissue enrichment analysis

We applied LDSC-SEG to assess tissues in which the heritability of ADs and IgAD is enriched. This analysis combined tissue-related gene expression data with GWAS summary statistics for each trait (Finucane et al. 2018). The 1000 Genomes Phase 3 European ancestry data was used as the reference panel for LD score calculation, restricting SNPs to those in HapMap3 to ensure data quality. Tissue-specific annotations were obtained from a pre-computed file that included 53 tissues from the GTEx project and 152 tissue types from the Franke lab dataset. Regression coefficient P values were derived from Z scores, with a threshold of P-value < 0.05 considered statistically significant for identifying enriched tissues across both conditions.

### Genome-wide gene-based analysis and transcriptome-wide association analysis

To identify global gene-level associations from GWAS summary statistics for each trait, we used MAGMA and TWAS. MAGMA was applied for gene-based analysis by aggregating SNP P-values using the SNP-wise mean model, with LD data obtained from the 1000 Genomes Phase 3 European reference panel (Leeuw et al. 2015). Transcriptome-wide association analysis (TWAS) was performed using the FUSION software, integrating GWAS summary statistics and pre-computed gene expression weights from GTEx cross-tissue expression data (Gusev et al. 2016). P-values from both analyses were corrected for multiple testing using the FDR, with statistical significance set below 0.01.

### Cell-type enrichment analysis

Single-cell RNA-seq data for peripheral blood mononuclear cells (PBMCs) were obtained from the OneK1K project (Yazar et al. 2022), while gut tissue data, including the colon, ileum, intestine, and lymph node, were sourced from the study by Elementai et al. (Elmentaite et al. 2021). Both datasets were retrieved from the CELLxGENE database, with cell type annotations and UMAP projections provided by the original authors (Abdulla et al. 2025). We performed expression-weighted cell-type enrichment (EWCE) analysis using the overlapping genes for each IgAD-AD pair to identify cell types significantly overexpressing genes shared between IgAD and ADs (Skene and Grant 2016). AUCell scores (Aibar et al. 2017) were used to quantify the enrichment of each overlapping gene set at the single-cell level, based on the relative ranking of gene expression within individual cells. These scores were subsequently visualized on the UMAP plots.

### Mendelian randomization analysis

To explore the potential causal relationship between IgAD and ADs, we conducted bidirectional two-sample Mendelian randomization (MR) using the R package “TwoSampleMR” (Hemani et al. 2018). Given the limited sample size in the original IgAD GWAS (Bronson et al. 2016), SNPs were selected as instrumental variables based on genome-wide significance thresholds of *P* < 1 × 10⁻⁶ for IgAD as the exposure and *P* < 5 × 10⁻⁸ for ADs as the exposure. LD clumping was performed with an r2 threshold of 0.001 and a 10,000 kb window, using the 1000 Genome Project European population as the reference, implemented via the R package “ieugwasr” (Rasteiro, G.H. 2025). To satisfy the exclusion-restriction assumption, SNPs weakly associated (p < 1 × 10^-5^) with outcome were excluded. F-statistics (F = beta.exposure^2^ /se.exposure^2^) were calculated to measure the strength of instrumental variables, retaining only those with F >10. Five main MR methods (Inverse variance weighted (IVW), weighted median, MR-Egger, weighted mode, and simple mode) were employed to test the causality between IgAD and ADs. Sensitivity analyses, including the leave-one-out sensitivity, pleiotropy, and heterogeneity tests, were performed. Outlier instrumental variables were identified and removed using leave-one-out analysis and the MR-PRESSO method (Verbanck et al. 2018).

## Results

### Genetic correlation between IgAD and autoimmunity

**Fig. 2:**
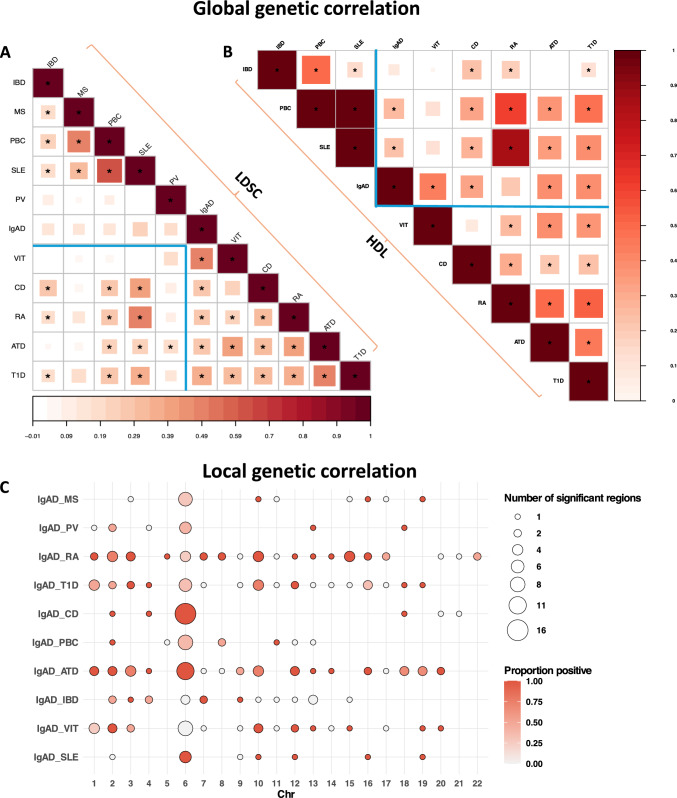
Global and local genetic correlations between IgAD and ADs. Global genetic correlations (r_g_) were estimated using LDSC **A** and HDL **B**. The color intensity and size of the squares indicate the magnitude of the r_g_. Asterisks represent statistical significance at a false discovery rate (FDR) level of 0.05 to account for multiple testing. LDSC produced genetic correlation estimates for IgAD and all 10 ADs, whereas HDL produced estimates for 8 diseases only. MS and PV were not included in the HDL results because usable heritability estimates could not be obtained, likely due to very low overlap between their summary statistics and the HDL reference panel. **C** Local genetic correlations were estimated using LAVA across independent genomic regions. Each bubble represents one IgAD–disease pair on a given chromosome. Bubble size indicates the number of regions with significant local genetic correlation after multiple-testing correction (FDR < 0.05), and bubble color indicates the proportion of those significant regions showing positive local genetic correlation. Global genetic correlation analyses using LDSC and HDL identified significant positive genetic correlations between IgAD and VIT, CD, RA, ATD, T1D, PBC and SLE. Local genetic correlation analysis identified 224 unique independent genomic regions with significant correlations between IgAD and all 10 ADs.

We analyzed the largest publicly available GWAS summary statistics for IgAD and 10 ADs, mainly based on studies of European ancestry, obtained from the GWAS Catalog (**Supplementary Table ****1**). We first conducted LDSC and HDL analyses to assess global genetic correlations between IgAD and these ADs. The LDSC results indicated significant positive genetic correlations (FDR < 0.05) between IgAD and VIT, CD, RA, ATD, and T1D (Fig. 2A). Similarly, the HDL results exhibited notable concordance with the LDSC findings, further implementing significant positive genetic correlations between IgAD and PBC as well as SLE (Fig. 2B).

To further investigate these genetic relationships, a local genetic correlation analysis was performed using LAVA to pinpoint independent genomic regions with significant correlations between IgAD and each autoimmune condition. LAVA identified 239 significant bivariate local genetic correlations (FDR < 0.05) between IgAD and the 10 ADs, spanning 224 unique genome regions (Fig. 2C). Of these, 147 (62%) were positive while 92 were negative correlations. Notably, the proportion of regions with significant negative genetic correlations exceeded 60% for IgAD with IBD, MS, and PBC. Chromosome 6 was observed to harbor the highest number of significant correlations, largely driven by strong signals in the HLA regions associated with both IgAD and ADs.

### Cross-trait genetic association analysis to identify novel and shared genetic loci between IgAD and autoimmunity

**Fig. 3:**
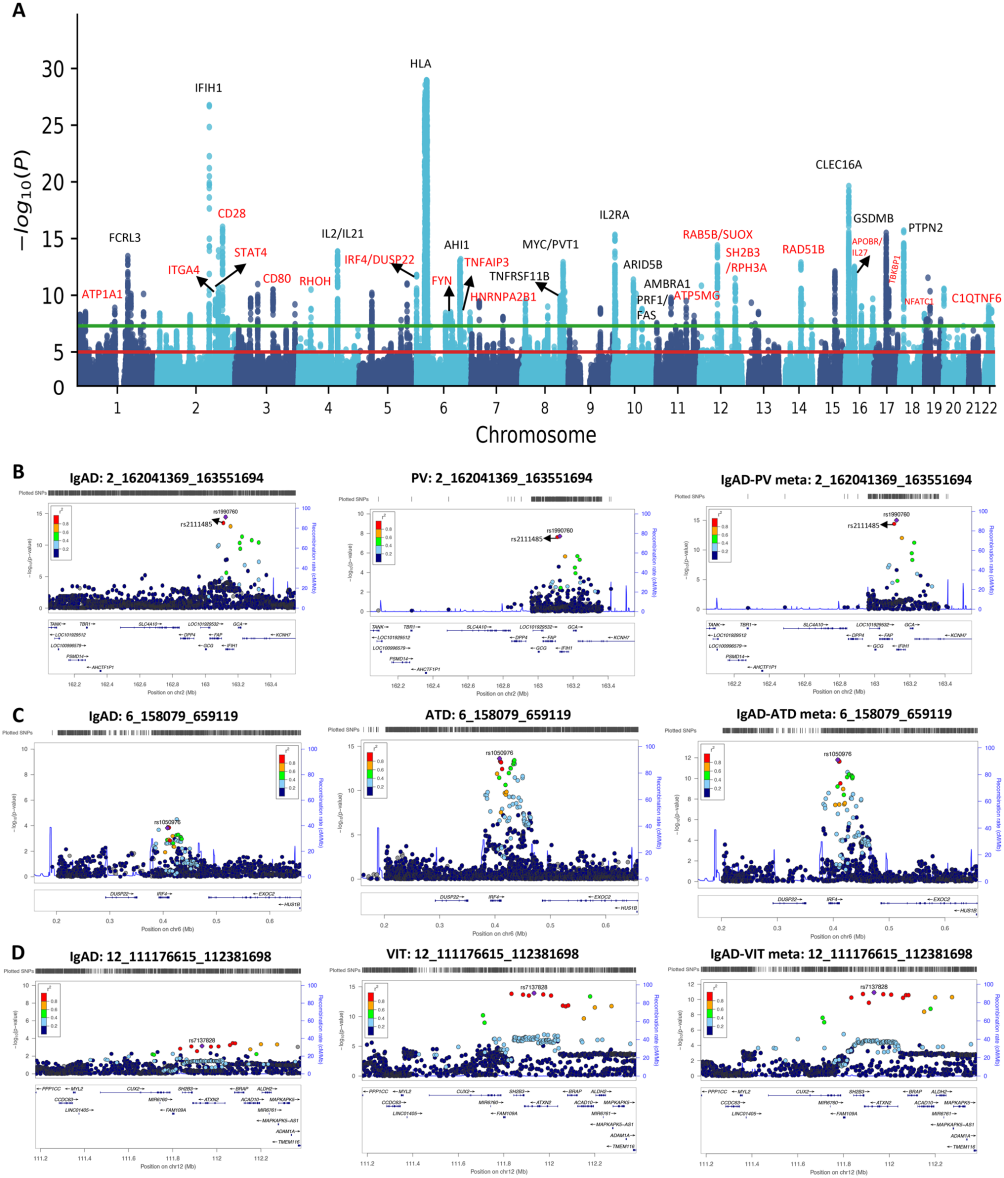
Cross-trait genetic association between IgAD and autoimmunity. **A** Manhattan plot of the cross-trait GWAS meta-analysis between IgAD and ADs. The x-axis represents the chromosomal position, and the y-axis represents the −log10 P value from PLACO. The green and red horizontal lines indicate the genome-wide significant thresholds of 5 × 10^-8^ and 1 × 10^-5^, respectively. Target genes for lead variants at each locus were annotated using the L2G and V2G pipelines from the Open Targets Platform. All 51 unique genetic loci identified in the analysis are listed in Supplementary Table 2. To improve readability, only loci shared by IgAD with at least two ADs or loci supported by colocalization analysis were labeled with gene names. Gene names shown in black indicate loci previously associated with IgAD, whereas gene names shown in red indicate loci newly identified for IgAD through our cross-trait analysis. **B** LocusZoom plots illustrate the original GWAS study of IgAD and PV, and the cross-trait meta-analysis of IgAD-PV in the locus of chr2:162,041,369-163,551,694 (*IFIH1*). **C** LocusZoom plots illustrate the original GWAS study of IgAD and ATD, and the cross-trait meta-analysis of IgAD-ATD in the locus of chr6:158,079-659,119 (*IRF4*). **D** LocusZoom plots illustrate the original GWAS study of IgAD and VIT, and the cross-trait meta-analysis of IgAD-VIT in the locus of chr12:111,176,615-112,381,698 (*SH2B3*).

Given that both local and global genetic correlation findings underscored a substantial shared genetic foundation between IgAD and ADs, we conducted cross-trait meta-analyses using GWAS summary statistics to systematically explore pleiotropic loci shared between each IgAD-AD pair using PLACO and MTAG. For each IgAD–AD pair, independent SNPs were identified from the cross-trait meta-analysis results using the PLINK clumping procedure. Independent loci were then defined by extending ±250 kb around each lead SNP, and overlapping regions were merged into a single locus. After this step, loci identified across all IgAD–AD pairs were further consolidated into distinct genomic loci by merging overlapping boundaries across pairs. The combined results of these analyses are illustrated in the Manhattan plot (Fig. 3A). Additionally, the original IgAD GWAS Manhattan plot is shown in Supplementary Figure A, while the Manhattan plots for each IgAD-AD GWAS meta-analysis are displayed in Supplementary Figure C. SNPs within the MHC region were excluded from downstream analyses to minimize the potential confounding effect. Across all pairwise analyses, we identified 51 unique genetic loci associated with IgAD at the genome-wide significance level (P_PLACO_ < 5 × 10^-8^ and P_MTAG-IgAD_ < 1 × 10^-5^), corresponding to 117 unique lead SNPs and 80 signals (r2 > 0.2 within a signal) (Figure 3A, Supplementary Figure 1B, Supplementary Table 2). Among these, most pleiotropic loci were observed in the IgAD-ATD pair (31 loci) and the IgAD-T1D pair (29 loci). Notably, 31 of these 51 loci were novel for IgAD (Supplementary Table 2).

We performed colocalization analyses for each pleiotropic locus to further validate shared genetic loci using the HyPrColoc and Coloc methods. These analyses identified 33 loci with evidence of colocalization (posterior probability (PP) from HyPrColoc or PP.H4 from Coloc > 0.5) (Table 1, Supplementary Table 2). Among these, 19 loci were novel for IgAD (Table 1), suggesting the widespread distribution of shared genomic risk loci between IgAD and ADs.

**Table 1:** Novel genetic loci associated with IgAD supported by Colocalization

	loci	Locus boundary(GRCH37)	Lead SNP for trait pairs	CHR:BP:A1:A2(GRCH37)	PLACO P-value	MTAG_IgAD P-value	Signals within locus (R2>0.2 within a signal)	Hyprcoloc(PP)	Coloc (PP.H4)	FUMA-nearestGene	FUMA-func Lead_snp	V2G top_gene
IgAD_SLE	1	1:116799215-117299215	rs10924105	1-117049215-C-T	4.66E-08	3.97E-06	rs10924105		0.71	RP5-1086K13.1	Intergenic	ATP1A1
IgAD_CD	2	2:181789477-182289477	rs6735551	2-182039477-C-T	2.49E-10	3.42E-07	rs6735551	0.8635	0.95	AC104820.2	ncRNA_intronic	ITGA4
IgAD_ATD	3	2:191673026-192232205	rs4341966	2-191982205-T-G	2.58E-09	1.26E-08	rs4341966	0.7755	0.84	STAT4	Intronic	STAT4
IgAD_ATD			rs1818625	2-191923026-C-T	2.03E-11	2.76E-10	rs1818625;rs10174238			STAT4	Intronic	STAT4
IgAD_T1D			rs10174238	2-191973034-A-G	4.24E-09	5.08E-07				STAT4	Intronic	STAT4
IgAD_CD	4	2:204362058-204884730	rs7426056	2-204612058-G-A	2.83E-10	3.64E-07	rs7426056;rs231389	0.8875	0.95	CD28	Intergenic	CD28
IgAD_T1D			rs231389	2-204634730-T-C	1.32E-10	4.27E-08				NPM1P33	Intergenic	CD28
IgAD_VIT	5	3:119034642-119534642	rs11533815	3-119284642-C-T	3.23E-11	8.61E-06	rs11533815		0.58	CD80	Intergenic	CD80
IgAD_ATD	6	4:40057564-40557564	rs13136820	4-40307564-T-C	3.45E-10	6.55E-09	rs13136820			AC195454.1	Intergenic	RHOH
IgAD_VIT			rs13136820	4-40307564-T-C	3.17E-11	3.25E-06			0.92	AC195454.1	Intergenic	RHOH
IgAD_ATD	7	6:158079-659119	rs1050976	6-408079-T-C	1.63E-12	7.22E-11	rs1050976;rs9391997	0.8863	0.89	IRF4	UTR3	DUSP22
IgAD_T1D			rs9391997	6-409119-G-A	1.78E-12	8.08E-09			0.94	IRF4	UTR3	DUSP22
IgAD_T1D	8	6:111826512-112326512	rs9400496	6-112076512-A-C	1.99E-08	2.49E-07	rs9400496		0.74	FYN	intronic	FYN
IgAD_RA	9	6:137888945-138472588	rs55771473	6-138160784-A-G	1.45E-09	1.09E-07	rs4548024;rs55771473;rs17780429;rs112906219	0.519		RP11-356I2.4	ncRNA_intronic	TNFAIP3
IgAD_ATD			rs55771473	6-138160784-A-G	1.02E-10	1.00E-10				RP11-356I2.4	ncRNA_intronic	TNFAIP3
IgAD_T1D			rs4548024	6-138165744-C-T	6.68E-14	9.75E-12			0.99	RP11-356I2.4	ncRNA_intronic	TNFAIP3
IgAD_SLE			rs112906219	6-138212862-T-C	4.52E-10	7.05E-09				TNFAIP3	Intergenic	TNFAIP3
IgAD_MS			rs17780429	6-138222588-A-G	3.14E-08	6.25E-07			0.69	TNFAIP3	Intergenic	TNFAIP3
IgAD_PV			rs17780429	6-138222588-A-G	7.30E-09	5.50E-07				TNFAIP3	Intergenic	TNFAIP3
IgAD_ATD	10	7:25885404-26385404	rs35257100	7-26135404-T-C	3.50E-10	3.83E-09	rs35257100		0.96	AC004520.1	Intergenic	HNRNPA2B1
IgAD_ATD	11	8:133964204-134464204	rs7005834	8-134214204-T-C	4.54E-11	1.22E-09	rs7005834		0.85	WISP1	Intronic	CCN4
IgAD_T1D	12	11:118047391-118547391	rs4435039	11-118297391-G-A	2.04E-08	1.39E-06	rs4435039	0.5417	0.8	ATP5L	Intronic	ATP5MG
IgAD_T1D	13	12:111176615-112381698	rs632650	12-112131698-T-G	1.71E-08	4.84E-06	rs632650		0.79	ACAD10	Intronic	ALDH2
IgAD_ATD			rs632650	12-112131698-T-G	9.54E-09	4.29E-08				ACAD10	Intronic	ALDH2
IgAD_T1D			rs7968960	12-111426615-A-C	1.06E-08	2.62E-06	rs7137828;rs7968960;rs11065979		0.79	RP1-46F2.2	Intergenic	GPN3
IgAD_VIT			rs7137828	12-111932800-T-C	1.24E-11	7.04E-06			0.76	ATXN2	Intronic	SH2B3
IgAD_CD			rs11065979	12-112059557-T-C	2.92E-09	1.95E-06			0.8	BRAP	Intergenic	ALDH2
IgAD_ATD	14	12:112769409-113485274	rs10850062	12-113019409-G-T	7.25E-09	7.49E-09	rs10850062	0.9222	0.79	RPH3A	Intronic	HECTD4
IgAD_ATD			rs976702	12-113235274-G-A	3.17E-09	3.83E-09	rs976702;rs2384035			RPH3A	Intronic	RPH3A
IgAD_T1D			rs2384035	12-113209185-G-A	3.67E-08	7.38E-06			0.8	RPH3A	Intronic	OAS1
IgAD_ATD	15	14:68489301-69551874	rs8015139	14-68739301-T-G	1.27E-10	2.88E-09	rs8015139;rs3784099	0.6773		RAD51B	Intronic	ZFP36L1
IgAD_T1D			rs3784099	14-68749927-A-G	3.09E-09	5.70E-07				RAD51B	Intronic	ZFP36L1
IgAD_T1D	16	16:28235141-29139486	rs149299	16-28485141-C-T	3.72E-13	2.96E-09	rs149299	0.5824	0.66	CLN3:CLN3	Intronic	IL27
IgAD_T1D			rs151233	16-28506428-T-C	4.04E-13	3.46E-09	rs151233			CLN3:APOBR	Exonic	SULT1A1
IgAD_ATD			rs151233	16-28506428-T-C	2.08E-09	2.43E-08				CLN3:APOBR	Exonic	SULT1A1
IgAD_ATD	17	17:45554494-46054494	rs4794063	17-45804494-T-C	3.10E-11	5.86E-10	rs4794063		0.61	TBX21	Intergenic	TBKBP1
IgAD_ATD	18	18:76928302-77428302	rs8093850	18-77178302-G-A	4.61E-09	5.36E-08	rs8093850		0.67	NFATC1	Intronic	NFATC1
IgAD_T1D	19	22:37325469-37894115	rs112578407	22-37575469-A-G	3.44E-08	7.53E-06	rs112578407	0.9824		RP1-151B14.6	ncRNA_intronic	C1QTNF6

Among the 33 loci with evidence for colocalization, a known locus around *IFIH1* colocalized with the highest number of ADs (PP=0.69), including IgAD and ATD, VIT, IBD, PBC, PV, SLE, and T1D (Supplementary Table 2, Supplementary Figure 2A). LocusZoom plots in Fig. 3B illustrate examples of the original GWAS study of IgAD and PV, and our cross-trait meta-analysis of IgAD-PV in this locus. HyPrColoc and Coloc analyses prioritized rs1990760 as the candidate causal variant for this locus. rs1990760 (original IgAD GWAS p-value=3.72E-15) is a missense variant in *IFIH1* and is in strong LD with the intergenic variant rs2111485 (original IgAD GWAS p-value= 3.32E-14). Interestingly, the rs1990760-T acts as a risk allele for IgAD, ATD, RA, T1D, VIT, PV and SLE, but is protective for IBD (OR=0.94) and PBC (OR=0.92), as determined by evidence of effect direction between IgAD and these diseases using METAL (Table 2, Supplementary Table 2). Additional studies in the GWAS Catalog further supported this allele-specific effect pattern across different ADs (Table 2). Another IgAD-known locus, located downstream of *PVT1,* colocalized with IgAD and ATD, IBD and PBC (PP = 0.57) (Supplementary Figure B). Colocalization analysis prioritized rs72722767 as the candidate causal variant for this locus.

**Table 2:** Allele-specific effect pattern of rs1990760 across different ADs

	Trait pair	Lead SNP	CHR:BP:A1:A2	OR_IgAD (Orignal GWAS)	PVALUE_IgAD (Orignal GWAS)	OR_AD [95% CI] (Orignal GWAS)	Pvalue_AD (Orignal GWAS)	Direction_Metal	HetPVal_METAL	PLACO P-value	MTAG_IgAD P-value	LAVA r_g_	LAVA P_value
Corss-trait GWAS meta-analysis	IgAD_ATD	rs1990760	2-163124051-T-C	1.43	3.72E-15	1.07 [1.05-1.09]	1.10E-14	++	6.31E-10	7.24E-25	7.18E-23	0.38	7.51e-06
	IgAD_RA					1.03 [1.01–1.05]	0.00599	++	2.26e-12	1.25e-09	4.71e-14	0.20	0.16609
	IgAD_IBD					0.94 [0.92–0.97]	3.45E-06	+-	1.17E-18	5.14E-15	5.05E-12	-0.59	2.93e-08
	IgAD_PBC					0.92 [0.88–0.96]	0.000266	+-	4.41E-18	5.35E-13	1.66E-14	-0.67	0.00349
	IgAD_PV					1.21 [1.13-1.30]	1.95E-08	++	0.003206	9.36E-16	5.18E-14	0.78	1.48e-07
	IgAD_SLE					1.13 [1.08–1.18]	1.93E-08	++	3.68E-06	7.93E-19	2.61E-16	0.75	0.00017
	IgAD_T1D					1.13 [1.10–1.16]	1.30E-17	++	7.60E-07	1.74E-27	3.84E-22	0.46	3.99e-06

	Trait	Lead SNP	CHR:BP:A1:A2	GWAS P-VALUE	OR	95% CI	PUBMEDID	Study Accession
Additional Supported studies in the GWAS catalog	VIT	rs1990760	2-163124051-T-C	1.8e-08	1.27	[1.17-1.38]	22561518	GCST001509
	T1D			5.8e-17	1.17	[1.13-1.22]	25751624	GCST005536
	SLE			3e-7	1.17	[1.10–1.24]	19838195	GCST004867
	PV			1e-7	1.19	[1.12-1.28]	25903422	GCST002874
	PBC			0.00136	0.90	[0.84-0.96]	26394269	GCST003129
	IgAD			4e-15	1.43	[1.31–1.56]	27723758	GCST003814
	IBD			1e-9	0.91	[0.89-0.94]	37262302	GCST90301318
	ATD			1.3e-18	1.07	[1.06-1.09]	39067062	GCST90319320
	RA			2.2e-18	1.23	[1.16-1.30]	35507331	GCST90243956

Cross-trait meta-analyses improve the statistical power to detect modest genetic associations that may not reach genome-wide significance in a single-trait analysis. Among the 19 of 31 novel loci supported by colocalization, one example is a locus at 6p25.3, targeting *IRF4,* which colocalizes between IgAD, ATD and T1D (PP= 0.89) (Supplementary Figure C). LocusZoom plots in Fig. 3C illustrate the original GWAS study of IgAD and ATD, along with the cross-trait meta-analysis of IgAD-ATD at this locus. Colocalization analyses prioritized rs1050976 as the candidate causal variant for this locus. The p-value for this variant in the original IgAD GWAS was 0.00015, while the cross-trait meta-analysis yielded a much stronger p-value of 1.63E-12. According to the ImmuNexUT database, the risk allele rs1050976-T is associated with decreased expression of *IRF4* in myeloid dendritic cells, monocytes, unswitched memory B and NK cells.

Another noteworthy locus at chr12:111,176,615-112,381,698 colocalizes between IgAD, ATD, CD, T1D and VIT (Supplementary Figure D). LocusZoom plots in Fig. 3D show examples of the original GWAS study of IgAD and VIT, along with the cross-trait meta-analysis of IgAD-VIT at this locus. The lead SNP rs7137828 (original IgAD GWAS p-value = 0.00071, cross-trait GWAS p-value = 1.24E-11) resides in the intron of *ATXN2,* and the risk allele C is associated with increased expression of *SH2B3* and *TRAFD1* but decreased expression of *ALDH2* and *TMEM116* according to the eQTLGen database. Similarly, loci targeting *STAT4* (Supplementary Figure A) and *CD28* (Supplementary Figure B) were identified, with lead SNPs from the original IgAD GWAS being rs10174238 (p-value = 0.00018) and rs7426056 (p-value = 1.91E-05), respectively. These results underscore the potential of cross-trait meta-analyses and the integration of GWAS data from related traits to increase the power of detecting novel association loci.

### Functional annotation and shared function between IgAD and autoimmunity

Functional annotation was performed using FUMA on the 51 unique genetic loci associated with IgAD and autoimmunity. These loci correspond to 117 unique lead SNPs identified through cross-trait meta-analysis. The annotation revealed that 46% of lead SNPs were located in intronic regions while 35% were found in intergenic (Supplementary Figure A). Additionally, five SNPs were located in exonic regions, including two missense variants (rs1990760, rs35667974) in *IFIH1*, one missense variant (rs7522061) in *FCRL3*, one missense variant (rs35947132) in *PRF1* and one synonymous variant (rs151233) in *APOBR*. Notably, *APOBR* was identified as a candidate gene in the IgAD novel loci that colocalize between IgAD, ATD and T1D (PP= 0.58). The risk allele rs151233-T was associated with decreased expression of *APOBR* in CD8 naive and central memory T cells based on significant cis-eQTLs from the OneK1K study (Supplementary Table 3).

**Fig. 4:**
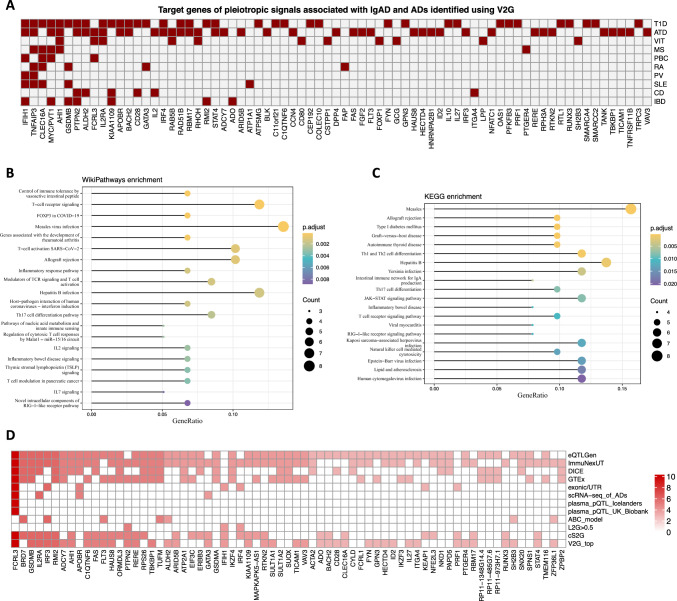
Target gene identification and pathway enrichment analysis on pleiotropic loci between IgAD and autoimmunity. **A** Target genes for each pleiotropic lead variant (117 unique lead SNPs) were prioritized using the L2G and V2G pipelines in the Open Targets Platform. For each variant, we selected the gene with the highest L2G score when available and also considered the gene with the highest V2G score. Using this approach, we identified 73 candidate genes shared between IgAD and ADs. **B, C** These 73 prioritized genes were used as the input gene set for the pathway enrichment analyses based on the WikiPathways (**B**)and KEGG **C**. **D** Heatmap showing target genes for each pleiotropic lead variant supported by at least three SNP-to-gene algorithms. The color intensity indicates the number of algorithms supporting each gene.

To prioritize target genes at these pleiotropic loci, we employed multiple SNP-to-gene mapping approaches, including physical mapping, cis-eQTL analysis, enhancer-gene linking, pQTLs evidence, and integrated SNP-to-gene linking strategies (**Methods**). Firstly, candidate genes were identified using the L2G and V2G pipeline in the Open Targets Platform, selecting genes with the maximum L2G (if available) and V2G scores for each variant. This analysis identified 73 candidate genes shared by IgAD and ADs. Among these, *IFIH1*, *TNFAIP3*, *CLEC16A*, *MYC/PVT1*, *AHI1*, *GSDMB* and *PTPN2* were shared across at least five diseases. Additionally, genes such as *TNFAIP3*, *BACH2*, *CD28*, *IRF4*, *RAB5B*, *STAT4*, *APOBR* and *RAD51B* were identified as candidate genes in the IgAD novel loci and shared among at least three diseases (Fig. 4A, Supplementary Table 3). We further conducted pathway enrichment analysis on these 73 genes as the input gene set to better understand their functional roles in IgAD and autoimmunity. The results revealed significant enrichment in immune-related pathways, including “Control of immune tolerance by vasoactive intestinal peptide” and “T cell receptor signaling” in the WikiPathways (Fig. 4B). Similarly, pathways such as immunological disease, T-cell differentiation, and “Intestinal immune network for IgA production” were enriched in the KEGG (Fig. 4C). Additional pathway enrichment analyses using the Reactome (Supplementary Figure B) and GO (Supplementary Figure C) further supported the involvement of these genes in immune-related pathways.

Next, we analyzed candidate genes for each SNP identified through 12 distinct algorithms, resulting in the nomination of 535 genes associated with IgAD and autoimmunity. Among these, 69 genes were supported by at least three algorithms (Fig. 4D**, ****Supplementary Table 3**). Within the novel loci, several genes were supported by at least five algorithms, including *IRF3*, *APOBR*, *C1QTNF6*, *RERE*, *RPS26* and *TBKBP1*. These findings reveal previously unknown genetic mechanisms underlying IgAD and provide new insights into its pathogenesis.

### Tissue and cell type enrichment of genetically overlapped genes between IgAD and autoimmunity

**Fig. 5:**
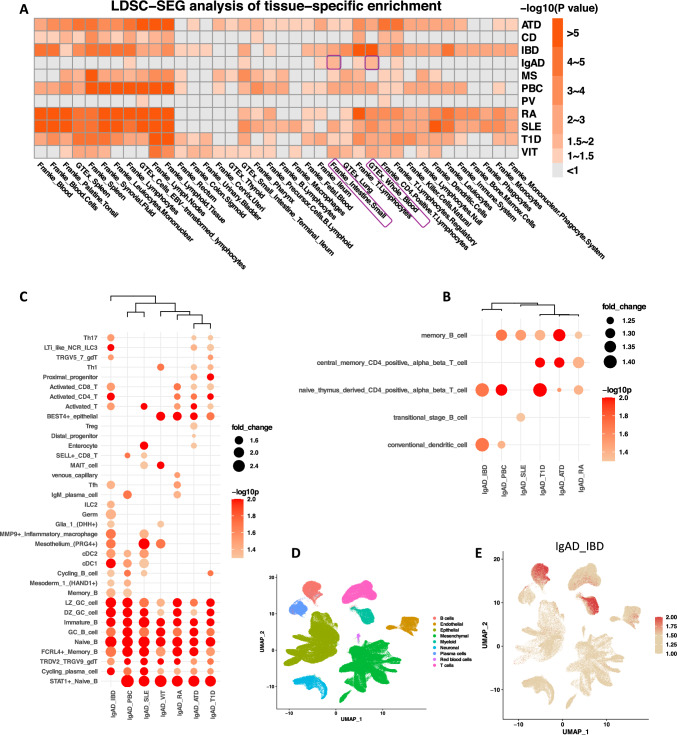
Tissue and cell-type enrichment between IgAD and autoimmunity. **A** Tissues significantly enriched (regression coefficient P-value < 0.05) in at least two diseases were identified using LDSC-SEG to assess tissue heritability of IgAD and ADs. **B, C** Cell-type enrichment analysis using single-cell RNA-seq data from PBMCs **B** and gut-derived tissues **C,** performed with expression-weighted cell-type enrichment (EWCE) based on overlapping genes for each IgAD-AD pair by MAGMA and TWAS. Dot size represents fold change, and dot color represents the enrichment significance as -log10 (P). **D** UMAP visualization of gut-derived cell types from the study by Elementai et al, with cells colored according to the high-level cell type annotations provided in the original dataset. **E** UMAP showing the distribution of the overlapping gene set identified for the IgAD–IBD pair in gut-derived cells. Color indicates the AUCell score, representing the aggregated expression enrichment of the shared gene set in each individual cell, with higher values indicating stronger enrichment.

We applied LDSC-SEG using the GTEx and Franke lab datasets to investigate tissue-specific heritability enrichment for IgAD and ADs. The analysis revealed that the heritability of IgAD was significantly enriched in the whole blood (GTEx), as well as in the small intestine, Ileum, and T Lymphocytes (Franke lab) (P < 0.05). For autoimmunity, the heritability of ATD, CD, IBD, PBC, RA, SLE, T1D and VIT was significantly enriched in GTEx or Franke whole blood. Additionally, the heritability of ATD, IBD, PBC, RA, SLE, T1D and VIT showed significant enrichment in the ileum and small intestine (P < 0.05). These findings underscore the common involvement of these tissues in the pathogenesis of both IgAD and ADs. All significant tissue enrichment results are presented in Supplementary Figure A, while Fig. 5A highlights tissues significantly enriched in at least two diseases.

To further explore globally shared genes between IgAD and ADs, we used MAGMA and TWAS to identify target genes based on GWAS summary statistics for each trait. This analysis identified a total of 338 genes significantly associated with IgAD (FDR < 0.01), of which 316 genes (93%) are shared with at least one AD (Supplementary Table 4), indicating a high degree of overlapping genetic associations between these two conditions. We also conducted pathway enrichment analysis for each trait using the genes identified by MAGMA and TWAS to understand their global functions (**Supplementary Figure ****5**). The enrichment showed similar major functional pathways across these conditions.

Subsequently, to investigate the cellular basis of shared genetic heritability, we applied expression-weighted cell-type enrichment (EWCE) to single-cell RNA-seq datasets, focusing on gut-derived tissues and PBMCs. Using the overlapping genes for each IgAD-AD pair identified through MAGMA and TWAS, our analysis consistently identified B cell subtypes as significantly enriched with shared genes across both tissues (Fig. 5B and C).

In the gut, B cell lineages—including naive B cells, immature B cells, plasma cells, germinal center B cells, and memory B cells—exhibited significant enrichment of overlapping genes between IgAD and ADs such as IBD, PBC, SLE, VIT, RA, ATD, and T1D (Fig. 5C). A UMAP visualization of gut cell types is shown in Fig. 5D**,** and an example of the enrichment of overlapping genes between IgAD and IBD is presented in Fig. 5E. Similarly, in whole blood, memory B cells and transitional stage B cells were significantly enriched with overlapping genes between IgAD and ADs, including PBC, SLE, T1D, ATD, and RA (Fig. 5B). A UMAP visualization of blood cell types is provided in Supplementary Figure B, and examples of the enrichment of overlapping genes between IgAD and SLE, and IgAD and ATD are shown in Supplementary Figures C and D, respectively.

Beyond B cells, myeloid cells in the gut showed significant enrichment of genes overlapping between IgAD and IBD, PBC, and SLE, while T cell subtypes—such as Th17 cells, activated CD4+ T cells, and activated CD8+ T cells—were enriched with genes overlapping between IgAD and IBD, RA, ATD, and T1D (Fig. 5C). In whole blood, central memory CD4+ T cells and naive thymus-derived CD4+ T cells exhibited enrichment with genes overlapping between IgAD and IBD, PBC, T1D, ATD, and RA. Additionally, conventional dendritic cells were enriched with genes overlapping between IgAD and IBD as well as PBC (Fig. 5B).

### The causal relationship between IgAD and autoimmunity

**Fig. 6:**
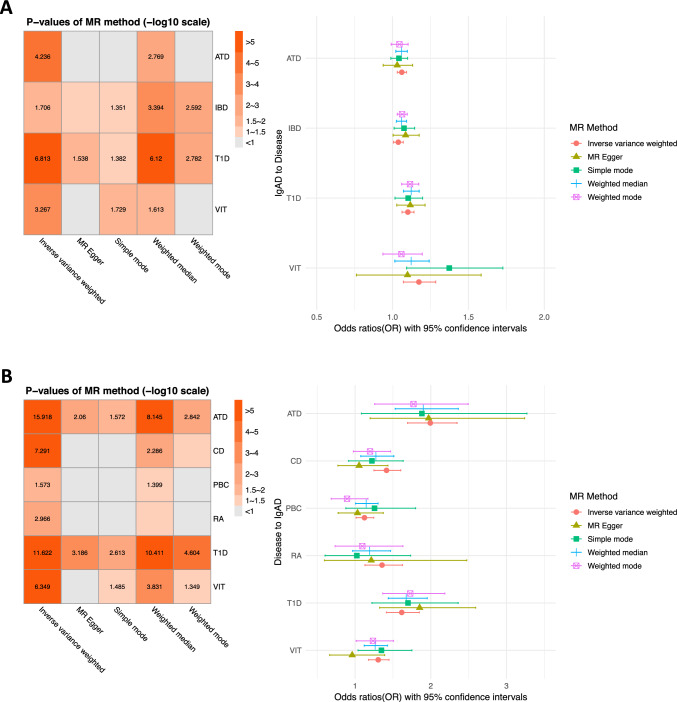
The causal relationship between IgAD and Autoimmunity. Causal associations from IgAD to ADs **A** and from ADs to IgAD **B** were examined using five Mendelian randomization methods.

We conducted bidirectional MR to explore the potential causal relationship between IgAD and ADs, employing five main MR methods to ensure the robustness of causal associations. Outlier SNPs were detected and removed using F-statistics > 10, leave-one-out analysis, and MR-PRESSO. The two-sample MR results revealed a bidirectional causal association between IgAD and several ADs. The analysis suggested that IgAD may increase the risk of T1D (IVW: OR=1.1, P=1.54e-07), IBD (IVW: OR=1.04, P=0.02), VIT (IVW: OR=1.17, P=0.00054) and ATD (IVW: OR=1.06, P=5.81e-05). The causal associations from IgAD to T1D and IBD were supported by five and four MR methods, respectively (Fig. 6A). Additional scatter and forest plots for the causal effect of IgAD on T1D and IBD are shown in Supplementary Figure . Conversely, several ADs appeared to increase the risk of IgAD, including ATD (IVW: OR=1.99, P=1.21e-16), T1D (IVW: OR=1.62, P=2.39e-12), VIT (IVW: OR=1.31, P=4.48e-07), CD (IVW: OR=1.42, P=5.11e-08), PBC (IVW: OR=1.12, P=0.03) and RA (IVW: OR=1.36, P=0.0011). The evidence for causal associations was observed for ATD, T1D, and VIT, supported by five, five, and four MR methods, respectively (Fig. 6B). Additional scatter and forest plots for the causal effects of ATD and T1D on IgAD are presented in Supplementary Figure .

## Discussion

Our study has unveiled a substantial overlap in genetic components between IgAD and ADs, providing genetic evidence to explain their frequent clinical co-occurrence. By elucidating the shared genetic architecture, we gain deeper insights into the functional pathways underlying both conditions, shedding light on their interconnected pathophysiology.

We demonstrated strong genetic correlations between IgAD and autoimmune conditions at both global and local levels, highlighting a heavily shared genetic architecture that underpins these diseases. Through cross-trait genetic association analyses using PLACO and MTAG, we identified 51 unique pleiotropic loci shared between IgAD and ADs. Additionally, colocalization analysis pinpointed 33 loci colocalizing across these conditions, emphasizing shared genetic mechanisms. One notable known locus is located in *IFIH1*, which is colocalized between IgAD and ATD, VIT, IBD, PBC, PV, SLE, and T1D. The candidate causal variant at this locus is rs1990760, a missense SNP that changes an amino acid substitution (Ala946Thr) in *IFIH1*. Previous reports have shown that the T allele of this variant is associated with increased type 1 interferon (IFN) levels (Robinson et al. 2011; Taylor et al. 2024). Our study observed that the T allele acts as a risk allele for IgAD and ATD, VIT, PV and SLE, but is protective for IBD and PBC. These observations align with findings from another GWAS study (Khrom et al. 2023), further supporting the pleiotropic effects of this variant. IFN levels play a pivotal regulatory role in immunoglobulin isotype selection (Thibault et al. 2009), and dysregulated IFN levels have been reported in IgAD patients (Shulzhenko et al. 2018). The changed IFN levels caused by rs1990760 may represent a shared factor contributing to the consistently dysregulated IFN responses observed in autoimmune patients (Wang et al. 2024) and the impaired IgA production characteristic of IgAD patients.

Among the 31 novel loci identified in our study, several intriguing candidate genes were mapped, including *TNFAIP3*, *BACH2*, *CD28*, *IRF4*, *RAB5B*, *STAT4*, *APOBR*, *RAD51B, SH2B3, RPH3A* and *C1QTNF6*. Notably, the locus targeting *IRF4* colocalizes between IgAD and ATD as well as T1D. *IRF4* is a key regulator of immunity, crucial for B cell differentiation and the facilitation of immunoglobulin isotype switching and secretion (Maffei et al. 2023). Similar mechanisms may also underlie the *STAT4* locus, which colocalizes between IgAD, ATD and T1D. The lead SNP rs10174238 is located in the intron of *STAT4*, a gene that plays a critical role in immune cell development and immunoglobulin production. Dysregulation of STAT4 could result in impaired IgA production and disruptions in immune regulation (Dong et al. 2021). Another notable novel locus, located on chr12:111,176,615-112,381,698, colocalizes between IgAD and ATD, CD, T1D and VIT. The lead SNP rs7137828-C is associated with increased expression of *SH2B3* and *TRAFD1* according to the eQTLGen database. *SH2B3* has been implicated in several ADs and is a key negative regulator of cytokine and growth factor receptor signaling. Its role in limiting the number of immature and transitional B cells may be particularly relevant in the context of IgA production and immune dysregulation (Zhang et al. 2024). Through these findings, our meta-analysis approach to IgAD and autoimmunity highlights how genetic variants affecting key immune regulators, such as *IRF4* and *STAT4*, disrupt normal immune processes. These results provide deeper insights into the common mechanisms driving both IgAD and autoimmunity. This knowledge not only enhances our understanding of IgAD but also highlights potential therapeutic targets for modulating immune function in affected individuals.

Pathway analysis revealed that the target genes of pleiotropic loci, identified using L2G and V2G, are enriched in pathways involved in the control of immune tolerance by vasoactive intestinal peptide (VIP), Intestinal immune network for IgA production, immunological disease, and T-cell differentiation. These findings highlight the shared pathogenic mechanisms underlying IgAD and autoimmunity. Dysregulation of these pathways disrupts immune tolerance, impairs IgA production, and promotes chronic inflammation, thereby contributing to the development of both conditions. For instance, alterations in T cell receptor signaling and Th17 differentiation are known to result in defective immune regulation and impaired mucosal defense. Simultaneously, disruptions in the intestinal immune network for IgA production can reduce IgA levels, compromising mucosal immunity (Zhou et al. 2025). Additionally, impaired VIP-mediated immune tolerance may exacerbate inflammatory responses (Villanueva-Romero et al. 2018). These findings underscore the interconnectedness of immune pathways in IgAD and ADs, revealing potential therapeutic targets to restore immune balance and mitigate disease progression.

We employed tissue enrichment analysis and identified the blood and gut as the primary tissues enriched with the heritability of both autoimmunity and IgAD. This finding aligns with the observation that approximately one-third of individuals with IgAD experience recurrent mucosal infections in the gut, underscoring the critical role of IgA antibodies in maintaining mucosal immunity (Moll et al. 2021). By leveraging single-cell data from blood and gut to investigate specific cell types associated with the overlapping genes for each IgAD-AD pair, we observed significant enrichment of shared genes in B cells across both the gut and PBMCs. This suggests that dysregulation of B cell function may play a pivotal role in the pathogenesis of both autoimmunity and IgAD. Previous studies have indicated that defects in memory B cells may contribute to impaired IgA production in these conditions (Grosserichter-Wagener et al. 2020). Additionally, dysregulated transitional B cells and abnormalities in Toll-like receptor functions have been reported in IgAD patients (Lemarquis et al. 2018), further supporting the hypothesis that defective B cell development and function represent a shared pathogenic mechanism underlying both diseases.

Furthermore, we identified specific T cell subtypes enriched with genes overlapping between IgAD and autoimmunity. Previous studies have also demonstrated the presence of dysfunctional T cells and abnormal levels of BAFF, APRIL, and TGF-β1 in IgAD patients (Grosserichter-Wagener et al. 2020). Collectively, these findings highlight the central roles of both B cells and T cells in the shared pathogenesis of IgAD and autoimmunity within the blood and gut, providing valuable insights into the cellular mechanisms underlying these conditions.

ADs are a common comorbidity in IgAD and may, in some cases, represent the first clinically apparent manifestation leading to diagnosis. IgAD is often recognized in childhood, whereas many ADs have a later and more variable age at onset. Mendelian randomization analysis revealed a bidirectional causal relationship between IgAD and ADs, particularly between IgAD and ATD, T1D as well as VIT, indicating that these traits may mutually influence one another at the level of genetic liability rather than through a simple unidirectional mechanism. However, this does not necessarily imply that ADs clinically cause IgAD after disease onset, especially given that IgAD is often recognized earlier in life. Instead, the reverse-direction effect may reflect shared inherited immune-regulatory mechanisms and overlapping susceptibility pathways that contribute to both conditions. Previous studies have shown that autoimmunity and immunodeficiency are closely interconnected. Immune dysregulation may contribute both to autoimmune manifestations and to impaired immune function, whereas immune defects may disrupt the induction or maintenance of self-tolerance, thereby promoting autoimmunity (Sogkas et al. 2021). Thus, the observed bidirectional association likely reflects an intertwined pathogenesis and shared biological pathways underlying both IgAD and ADs. Elucidating the shared genetic architecture and molecular signatures of both conditions may help clarify the mechanisms driving their co-occurrence and inform future therapeutic strategies.

Our study has several limitations. First, given that only one set of available IgAD summary statistics exists for the European population, further replication studies in populations of diverse ancestries are essential to validate our findings. Second, the pathways and cell types identified in this study require future experimental validation to confirm their specific role in the pathogenesis of both IgAD and autoimmunity. Third, both the PLACO and MTAG approaches used in this study rely on the presence of genetic variants in both GWAS datasets for each IgAD-AD pair. As a result, some variants retained only in one dataset may not be included in the cross-trait GWAS meta-analysis, potentially leading to the exclusion of relevant loci. Fourth, there was substantial imbalance in sample size between the IgAD GWAS and several AD GWAS datasets. Although a strong association in only one disease would not automatically produce a significant shared signal, this imbalance may still affect interpretation. The relatively small IgAD GWAS likely reduced power to detect true shared associations. Moreover, MTAG leverages genetic covariance across traits and may amplify signals from the better-powered trait, such that some loci appear more significant even when direct support from the IgAD GWAS is limited. By contrast, PLACO is less susceptible to identifying loci based solely on a single-trait effect, but limited power in IgAD may still reduce sensitivity to detect true pleiotropy. Despite these limitations, cross-trait meta-analysis can improve power to detect modest shared associations that may not reach genome-wide significance in single-trait analyses. Finally, an important limitation of this study is that all analyses were based on GWAS summary statistics, which do not permit direct inference of classical HLA haplotypes or conditioning on major HLA class II risk haplotypes. Given the strong contribution of the HLA region to many ADs, future studies using individual-level genotype or sequencing data will be needed to determine whether the associations identified here act in an HLA haplotype-specific manner and to better resolve the mechanisms underlying these shared genetic signals.

## Conclusions

This study identified 51 shared genetic loci between IgAD and ADs. Notably, 19 out of 31 novel IgAD loci are supported by colocalization analysis, offering new insights into potential mechanisms underlying the comorbidity of IgAD and ADs. Shared pathways between IgAD and ADs include T-cell differentiation, immune tolerance regulated by vasoactive intestinal peptide, and the intestinal immune network for IgA production. Tissue and cell-type enrichment analyses revealed whole blood, intestine, and B cell subtypes as key contributors to both conditions. Additionally, Mendelian randomization analysis provided evidence of intertwined causal relationships between IgAD and specific ADs. The study highlights a shared genetic architecture and common pathogenic mechanisms between IgAD and autoimmunity. The enrichment of heritability in immune-related tissues and B cell subtypes underscores their pivotal role in disease pathogenesis, guiding future mechanistic studies, clinical research, and drug development.

## Supplementary Information

Below is the link to the electronic supplementary material.

Supplementary Figures (PDF 5665 KB)

Supplementary Tables (XLSX 280 KB)

## Data Availability

Data are available in public, open-access repositories corresponding to the original studies (e.g., GWAS catalog). Additional data that support the findings of this study are available within the paper and its Supplementary Information, or from the corresponding author upon reasonable request.

## Abbreviations

ADs: Autoimmune diseases
ATD: Autoimmune thyroid disease
CD: Celiac disease
eQTL: Expression quantitative trait loci
EWCE: Expression-weighted cell-type enrichment
FDR: False discovery rate
GWAS: Genome-wide association studies
HDL: High-definition likelihood
IBD: Inflammatory bowel disease
IgAD: Immunoglobulin A deficiency
IVW: Inverse variance weighted
LD: Linkage disequilibrium
LDSC: Linkage disequilibrium score regression
MAF: Minor allele frequency
MHC: Major histocompatibility complex
MR: Mendelian randomization
MS: Multiple sclerosis
MTAG: Multi-trait analysis of GWAS
PBC: Primary biliary cirrhosis
PBMC: Peripheral blood mononuclear cells
PLACO: Pleiotropic analysis under composite null
PP: Posterior probability
pQTL: Protein quantitative trait loci
PV: Common Psoriasis
RA: Rheumatoid arthritis
scRNA-seq: Single-cell RNA sequencing
SLE: Systemic lupus erythematosus
T1D: Type 1 diabetes
TWAS: Transcriptome-wide association analysis
VIT: Vitiligo
