# Supplementary material for: Investigating the shared genetic architecture between selective immunoglobulin A deficiency and autoimmune diseases
Source: Hum Genet. 2026 Jun 22;145(1):53. doi: 10.1007/s00439-026-02850-5 (PMC13287149; doi:10.1007/s00439-026-02850-5)
Supplement: Supplementary file 1 — Supplementary Figures (PDF 5665 KB) [file 439_2026_2850_MOESM1_ESM.pdf]

Investigating the shared genetic architecture between selective immunoglobulin A deficiency and autoimmune diseases

Xiao Dang[1*], Frank Qingyun Wang[1*], Caicai Zhang[1*], Huidong Su[1], Yao Lei[1], Jing Yang[1], Yu Lung Lau[1], Wanling Yang[1#]

[1]Department of Paediatrics and Adolescent Medicine, The University of Hong Kong, Hong Kong, China

* These authors contributed equally to this work

# Correspondence should be addressed to Dr. Wanling Yang,

Department of Paediatrics and Adolescent Medicine, The University of Hong Kong, Email: yangwl@hku.hk

Supplementary Figures

Supplementary Figure 1: Manhattan plots illustrating **(A)** the original IgAD GWAS, **(B)** the cross-trait GWAS meta-analysis conducted using PLACO, combined results from IgAD and 10 ADs, and **(C)** the cross-trait GWAS meta-analysis results for each IgAD-AD pair obtained using PLACO.

Supplementary Figure 2: LocusZoom plots of several known genomic loci for IgAD and ADs from the original GWAS summary statistics, including chr2:162,041,369-163,551,694 (*IFIH1*) **(A)**, chr8:128,826,509-129,503,544 (*PVT1*) **(B)**.

Supplementary Figure 3: LocusZoom plots of several novel genomic loci for IgAD and ADs from the original GWAS summary statistics, including chr2:191,673,026-192,232,205 (*STAT4*) **(A)**, chr2:204,362,058-204,884,730 (*CD28*) **(B)**, chr6:158,079-659,119 (*IRF4*) **(C)**, and chr12:111,176,615-112,381,698 (*SH2B3*) **(D)**.

Supplementary Figure 4: **(A)** Distribution of functional consequences of lead SNPs in the genomic loci shared between IgAD and ADs. **(B, C)** Pathway enrichment analysis on target genes using the Reactome (**B**) and Gene Ontology (GO) database (**C**).

Supplementary Figure 5: Pathway enrichment analysis for each trait based on genes identified by MAGMA and TWAS using the KEGG **(A)**, WikiPathways **(B)**, Reactome (**C**), and Gene Ontology (GO) database (**D**).

Supplementary Figure 6: **(A)** Tissues significantly enriched (regression coefficient P-value < 0.05) using LDSC-SEG to assess tissue heritability of IgAD and ADs. **(B)** UMAP visualization of blood cell types from the OneK1K project, with cells colored according to the high-level cell type annotations provided in the original dataset. **(C)** Enriched cell types in blood using overlapping genes between IgAD and SLE. **(D)** Enriched cell types in blood using overlapping genes between IgAD and ATD. Color indicates the AUCell score, representing the aggregated expression enrichment of the shared gene set in each individual cell, with higher values indicating stronger enrichment.

Supplementary Figure 7: Scatter and forest plots for the causal effect of IgAD on IBD (**A & B**) and T1D (**C & D**).

Supplementary Figure 8: Scatter and forest plots for the causal effect of ATD (**A & B**) and T1D (**C & D**) on IgAD.

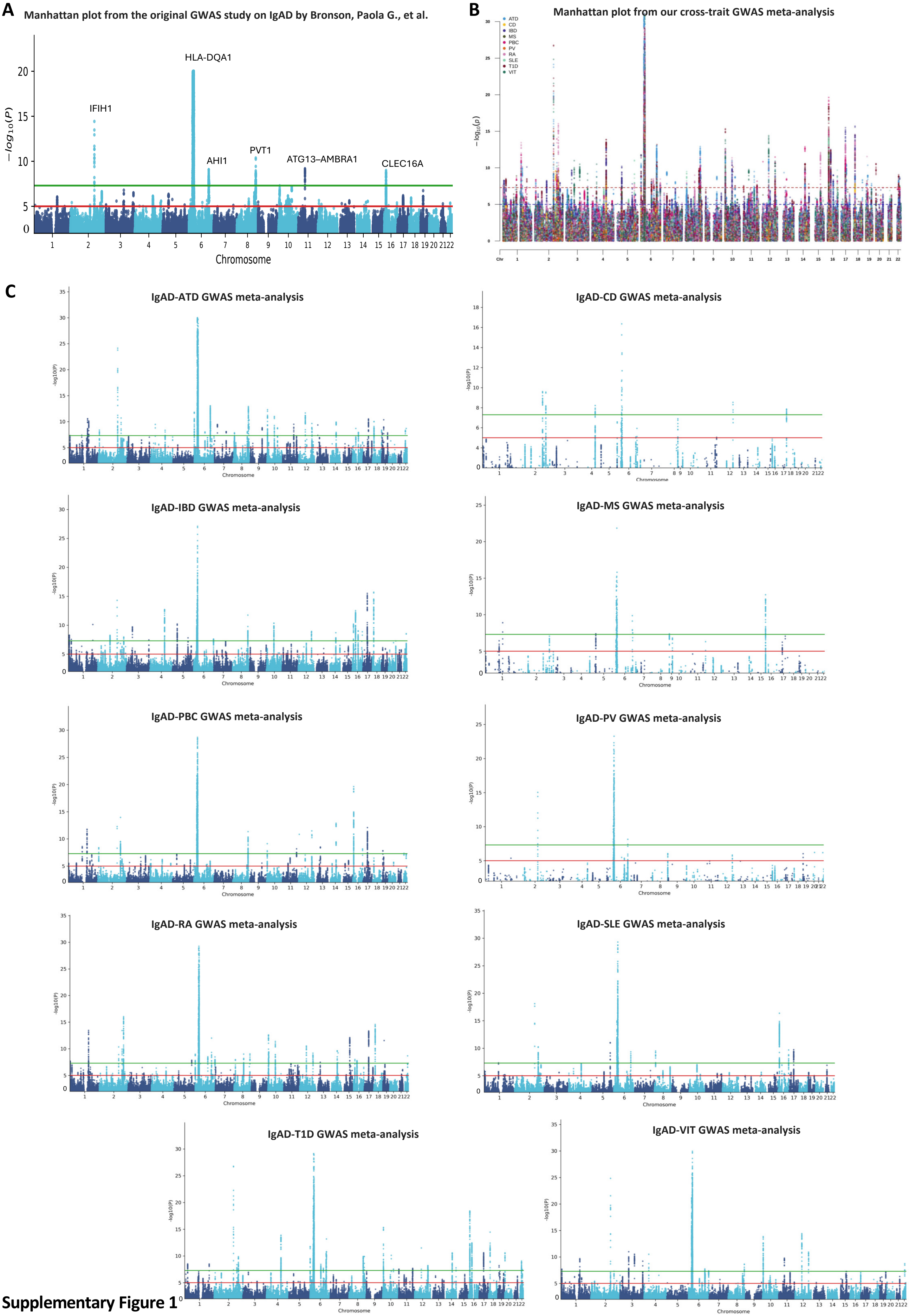

Supplementary Figure 1

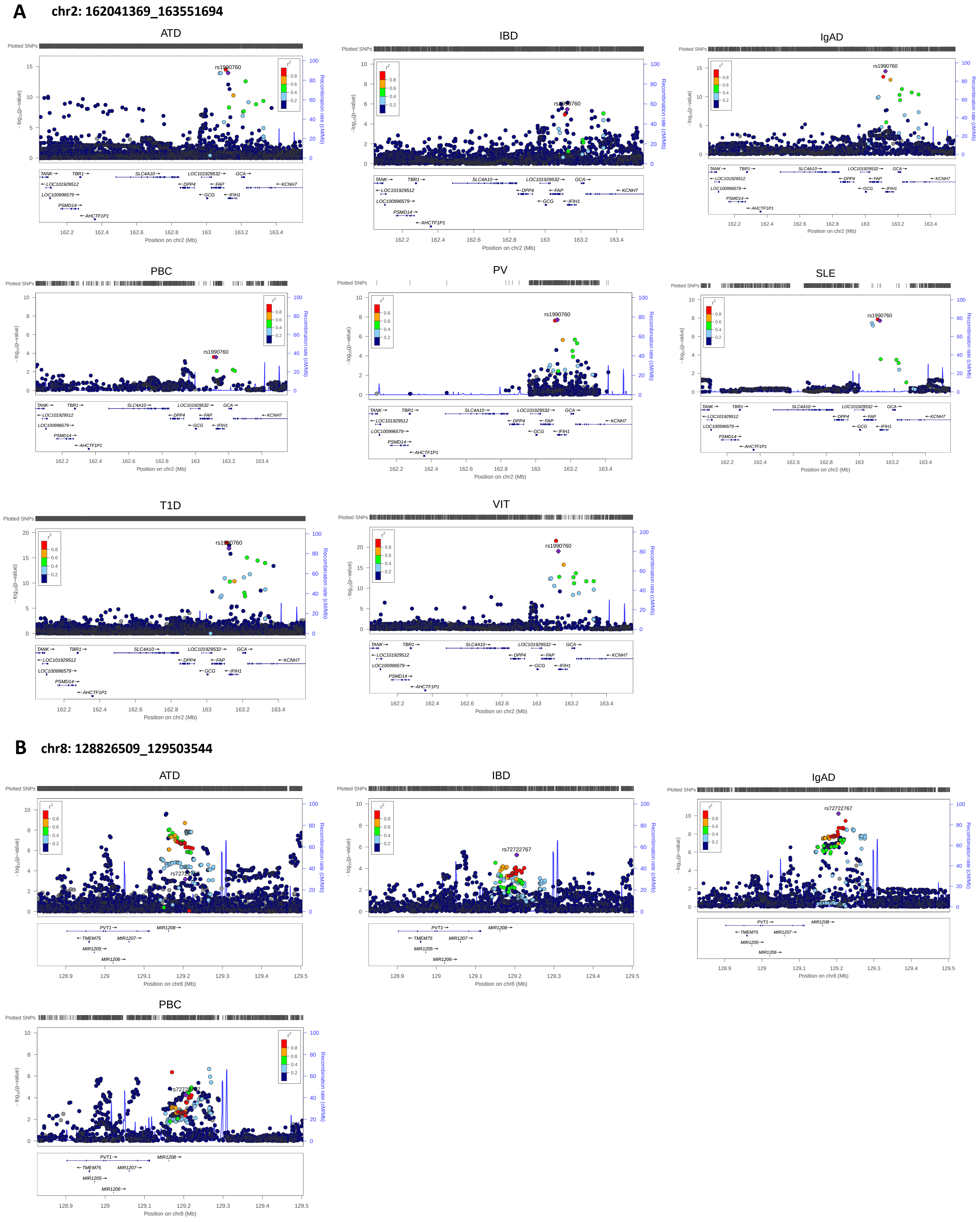

Supplementary Figure 2

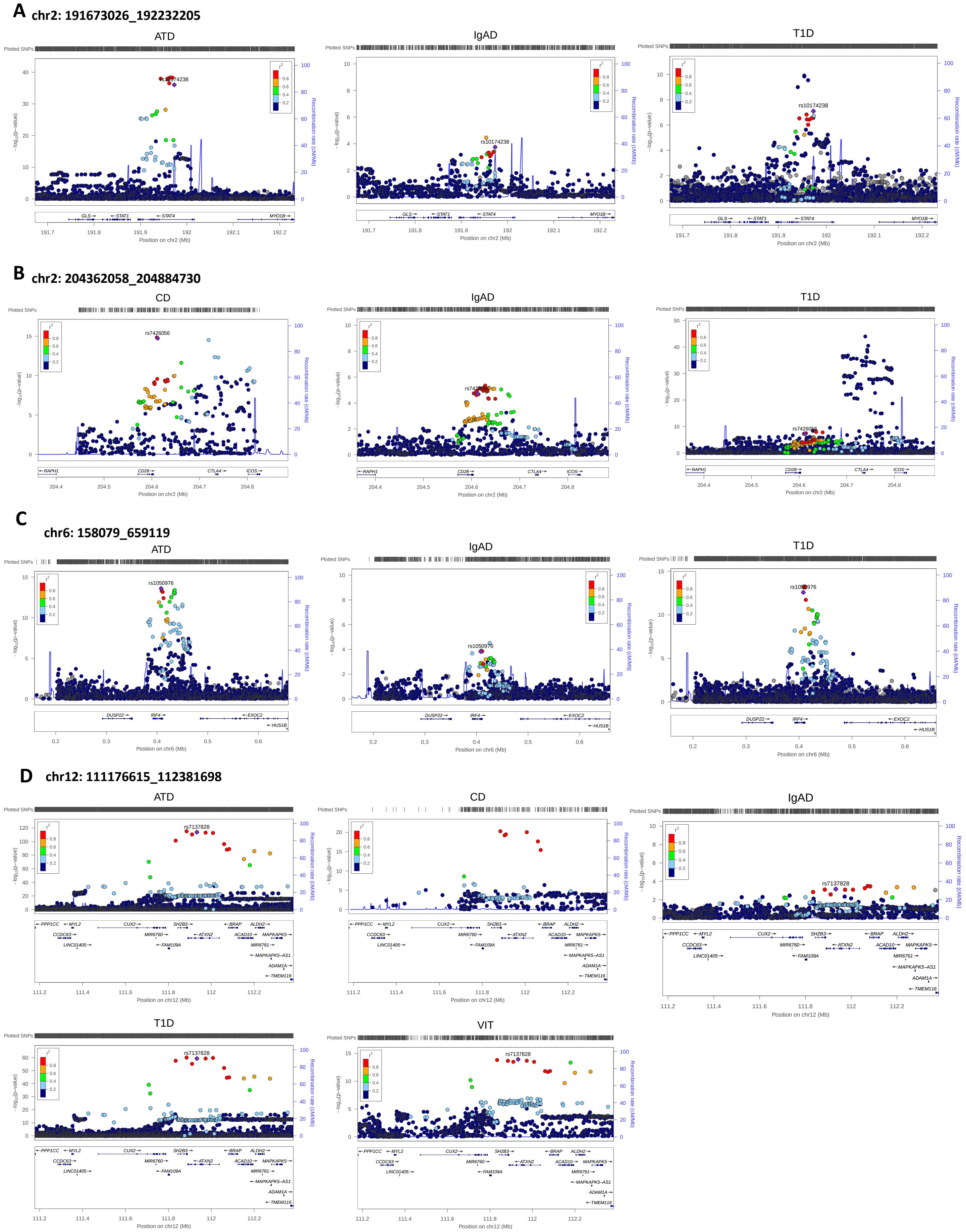

Supplementary Figure 3

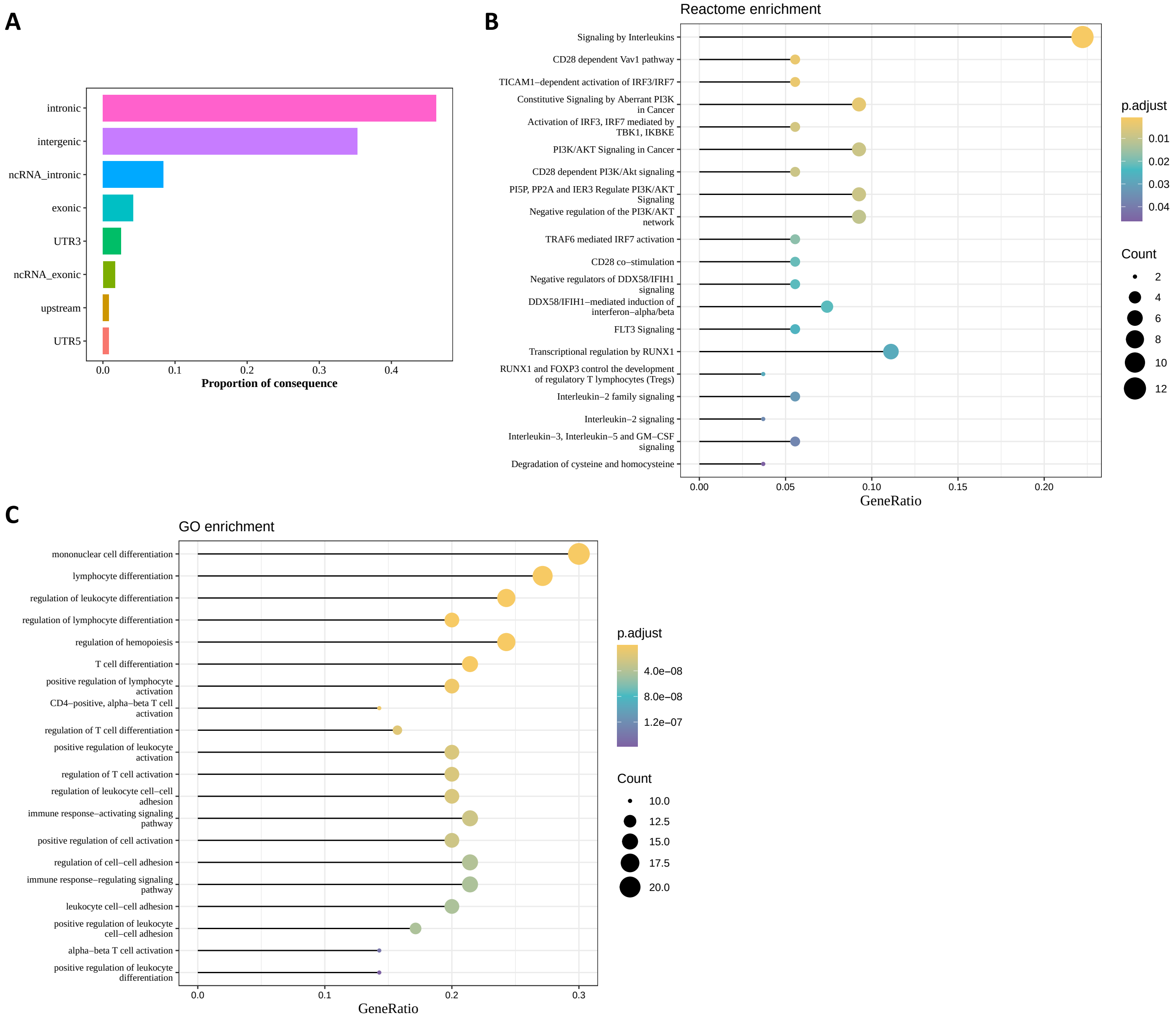

Supplementary Figure 4

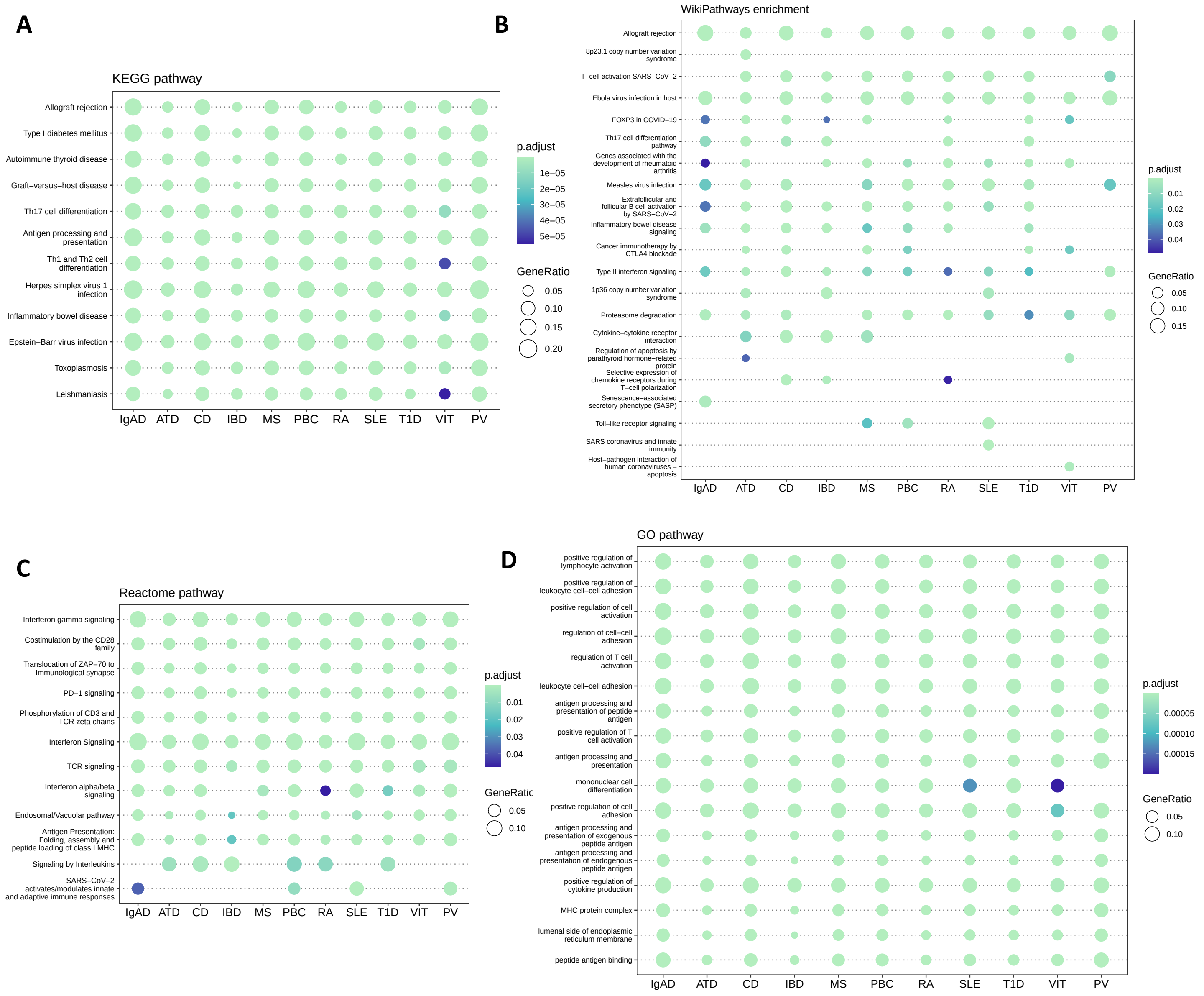

Supplementary Figure 5

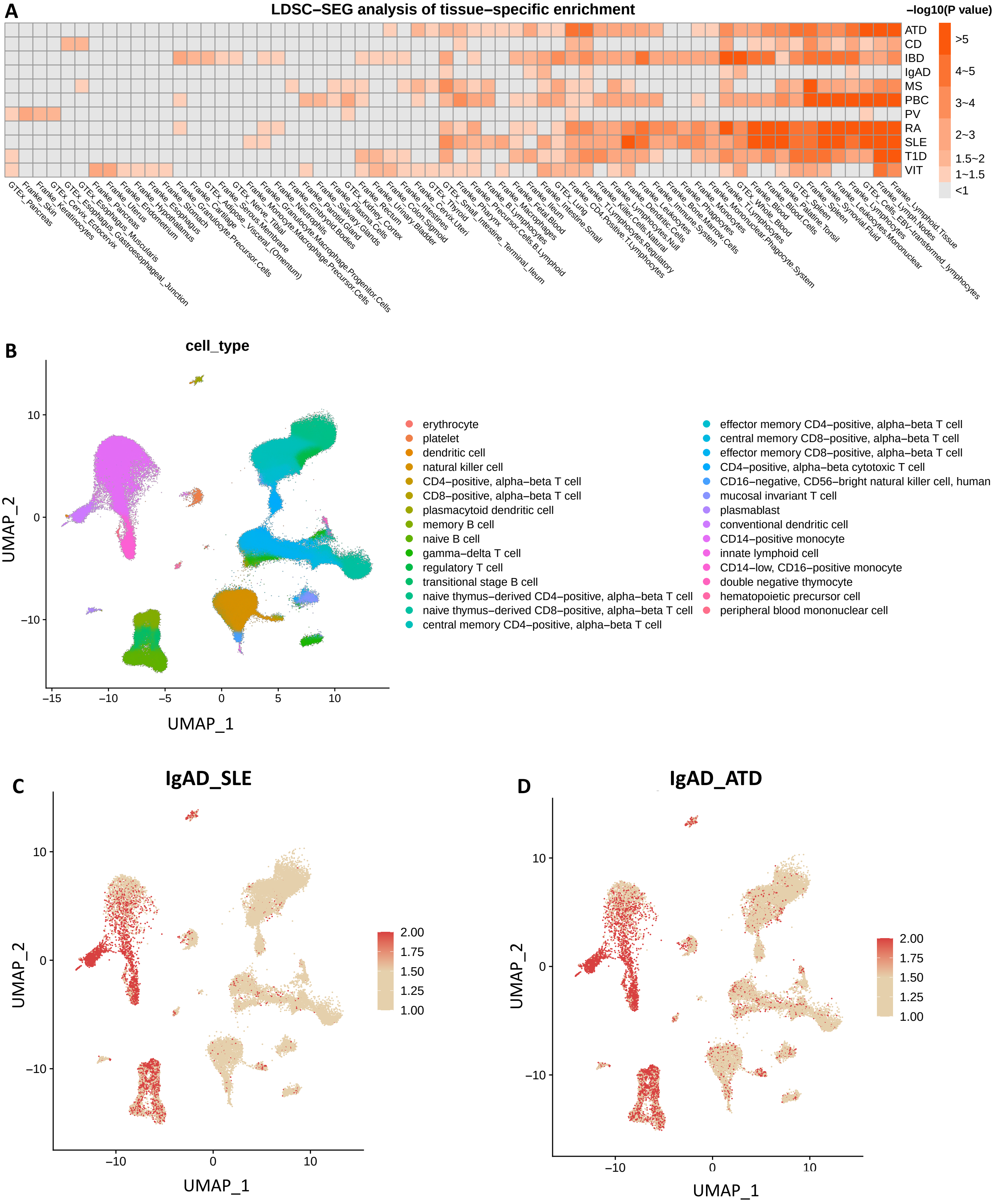

Supplementary Figure 6

IgAD to IBD

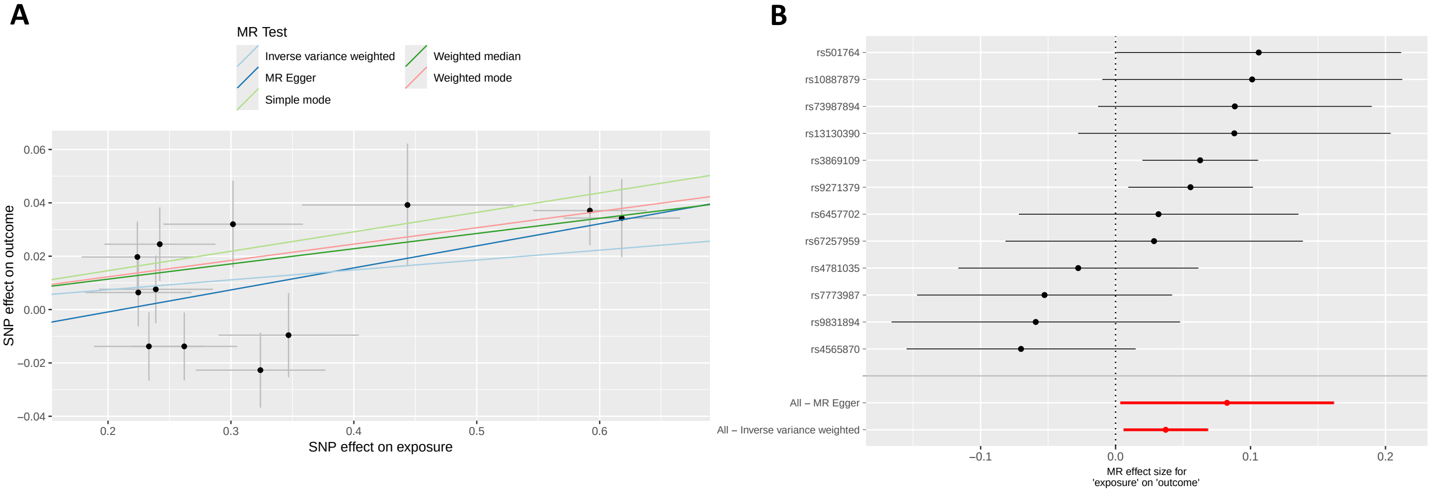

IgAD to T1D

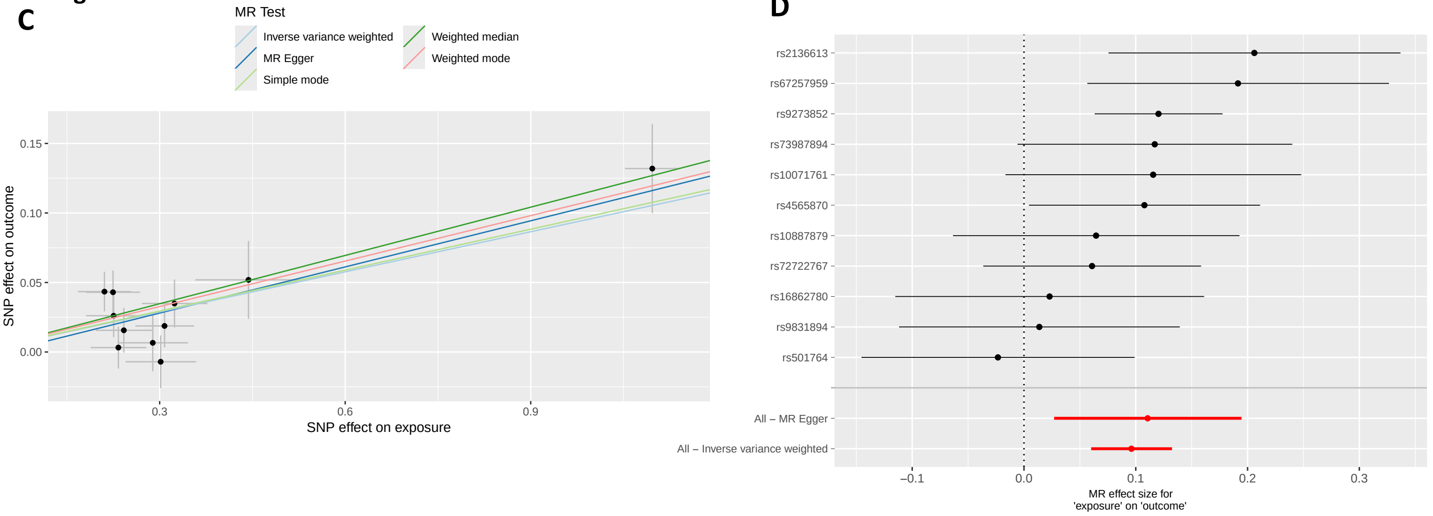

Supplementary Figure 7

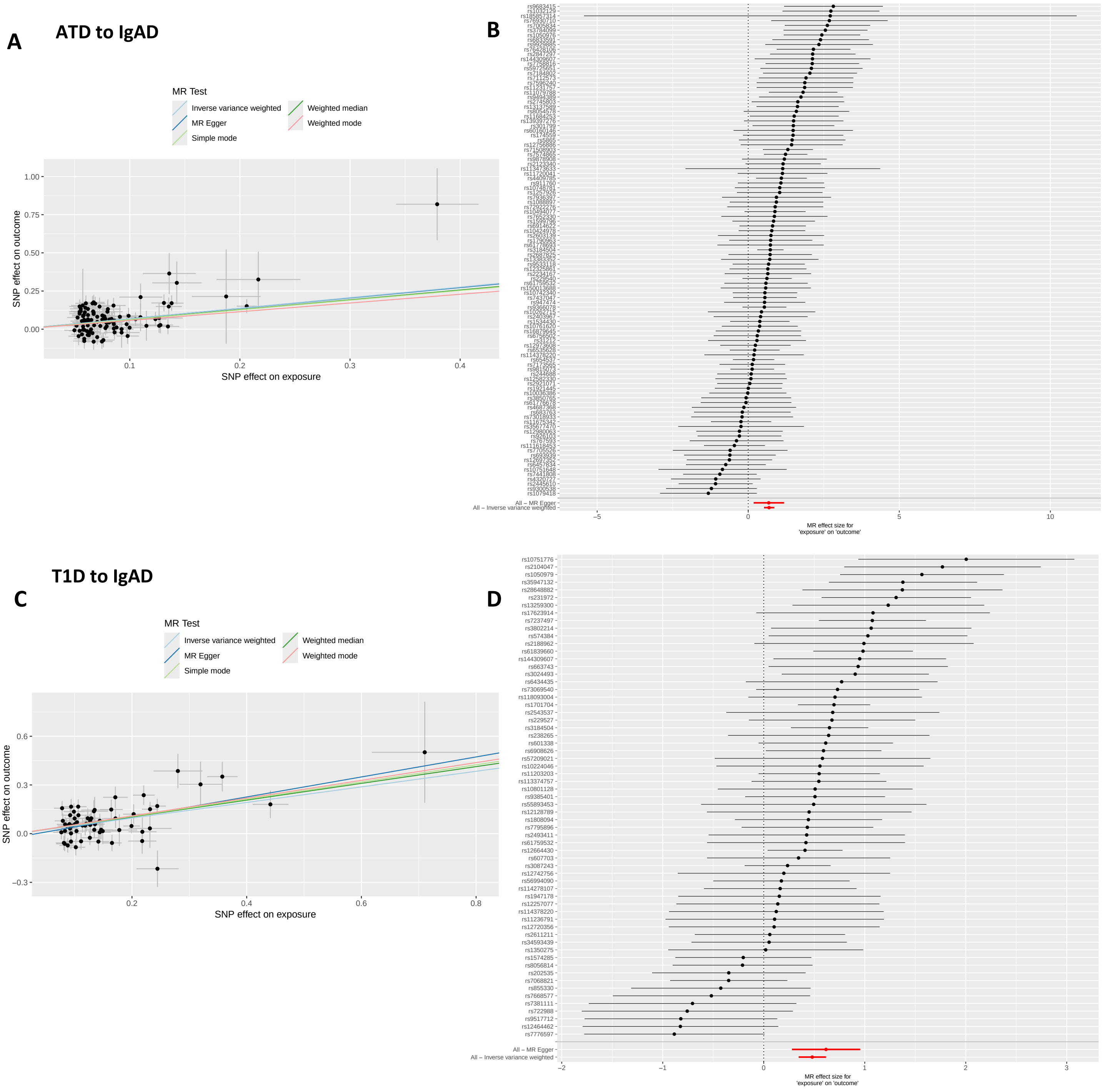

Supplementary Figure 8
